# Beneficial Use and Potential Effectiveness of Physical Activity in Managing Autism Spectrum Disorder

**DOI:** 10.3389/fnbeh.2020.587560

**Published:** 2020-10-22

**Authors:** Jessica Atef Nassef Sefen, Sabrina Al-Salmi, Zoya Shaikh, Jawaher Tariq AlMulhem, Ebrahim Rajab, Salim Fredericks

**Affiliations:** Royal College of Surgeons in Ireland (RCSI)-Bahrain, Al Muharraq, Bahrain

**Keywords:** early intervention, autism, autism spectral disorder (ASD), autism spectrum-disorder, physical activity, neurodeveloment, children with ASD, parental involvement

## Abstract

Autism Spectrum Disorder (ASD) is a neurodevelopmental condition characterized by poor social and communication skills. Therapeutic interventions are behavioral and educational—normally delivered as structured programs. Several well-established programs exist and most of them do not incorporate physical activity and exercise as core elements. Deficiencies in motor skills are associated with ASD and physical activity has been shown to reduce maladaptive behaviors with autistics. However, the notion of exercise being employed to manage autism is controversial. Meta-analysis and systematic reviews have concluded that physical activity has positive effects on social skills and behavior in young children and adolescents with autism. Activities such as martial arts have been singled out as being particularly beneficial. Established programs such as TEACCH have been successfully modified, as research trials, to be more physical activity-based and have shown positive results. Studies have also reinforced the importance of the role of parental involvement in delivering programs based on physical activity. There is a paucity of research evidence about the long-term effects of physical activity-based interventions. There is also disparity over the detailed nature of the activities and exercises that compose an effective program. Each person with autism has a highly individualized set of symptoms and characteristics for which highly individualized programs are warranted. This is especially true for physical activity programs.

## Introduction

Autism spectrum disorders (ASD) are neurodevelopmental conditions characterized by poor social communication and social interaction, as well as restricted repetitive patterns of behavior, interests, and activities (Levy et al., 2009). ASD is typically first recognized in early childhood (Christensen et al., 2016) and many of the available treatments are implemented as early interventions initiated during the pre-school years. There is currently no established curative treatment for ASD, and interventional approaches are behavioral and educational (Kaplan and McCracken, 2012; Medavarapu et al., 2019), and are normally delivered as structured programs.

Currently, there are two broad classes of ASD interventions; *comprehensive treatment models* which are structured programs, and *focused intervention practices* that only treat narrow behavioral symptoms (Wong et al., 2015). These focused single medication strategies include pharmaceuticals, nutritional supplements/special diets (e.g., melatonin, gluten-free, casein-free, vitamins, and minerals), and alternative or complementary medicine (e.g., chelation, neurofeedback, hyperbaric oxygen therapy, acupuncture, and stem cell therapy; Wong et al., 2015; Siniscalco et al., 2018; Medavarapu et al., 2019). Psychotropic drugs may also play a role in the management of some behaviors commonly associated with ASD, such as aggression, severe irritability, and hyperactivity. However, limited improvements have been reported with the use of psychopharmacologic agents to treat the core features of ASD (Kaplan and McCracken, 2012; DeFilippis and Wagner, 2016).

Early intervention programs address the broader aspects of communication and social skills. These are often delivered by multiple professionals including pediatricians, child psychiatrists, occupational therapists, speech therapists, psychologists, specialist-teachers, and parents. These teams contribute to educational and therapeutic services. Various treatment programs have become established which include Applied Behavioral Analysis (ABA), Early Intensive Behavioral Intervention (EIBI), Verbal Behavioral Intervention (VBI), Treatment and Education of Autistic and Related Communication-handicapped Children (TEACCH), Picture Exchange Communication System (PECS), speech therapy, and sensory integration therapy. Also added to this list are less conventional approaches such as horseback riding therapy, dolphin-assisted therapy, music therapy, and art therapy. This vast array of treatments, therapies, and programs have varying degrees of empirical support for their evidence base (Wong et al., 2015).

The use of physical exertion as a means of decreasing problem behaviors or increasing appropriate behaviors (exercise-therapy) is considered an evidence-based practice (Wong et al., 2015). Furthermore, research has revealed that disturbances and deficiencies in motor skills are commonly associated with ASD (Leary and Hill, 1996; Ghaziuddin and Butler, 1998; Ozonoff et al., 2008; Green et al., 2009). Given this evidence, exercise-therapy would seem an appropriate therapeutic strategy for improving the quality of life in ASD. This review discusses the benefit Physical Activity has on individuals with ASD and its potential effectiveness when employed as a tool to manage ASD. There is a paucity of studies that have examined PA among adults with ASD (Hillier et al., 2020), therefore the focal area of attention here is children and adolescents with ASD.

## The Benefits of Physical Activity in ASD

Physical activity (PA) is important for maintaining health within the general population. It reduces blood pressure, improves sleep quality and insulin sensitivity, and consequently lowers the risk of certain chronic conditions (Williamson and Pahor, 2010). Exercise induces the release of endorphins and monoamine neurotransmitters in the brain, thus mimicking the effects of antidepressants and making PA a viable alternative to drug treatments (Zhao and Chen, 2018). PA has been shown to improve motor skills and cognition in preschool children aged 4–6 years, specifically in the areas of attention, memory, behavior, and academic achievement (Lang et al., 2010; Zeng et al., 2017).

The patent health benefits of PA are also applicable to children with ASD. However, PA levels in children with ASD are lower than their typically developing (TD) counterparts (Macdonald et al., 2011; Hillier et al., 2020). This may be attributed to poor motor coordination and balance, often associated with ASD (Ghaziuddin and Butler, 1998; Green et al., 2009). These limit activity choices. Impairments in sensory, behavioral, and communication skills render participation in team activities challenging (Potvin et al., 2013). Hence, adolescents with ASD also engage in less PA relative to their TD counterparts (Srinivasan et al., 2014; Stanish et al., 2017).

Trials have been performed exploring the use of PA in ASD, many of which have endorsed the use of PA in the management of Autism. A systematic review by DeJesus et al. (2020) concluded that dance had a positive effect on ASD associated symptoms. These included improved: social involvement, behavior, communication skills, body awareness, and mental health. Meta-analysis has assessed PA interventions on youth with ASD across 29 studies and found an overall moderate positive effect (Healy et al., 2018). However, within the areas of movement (manipulative and locomotor skills, muscular strength, and endurance) moderate-to-large positive effects were found. Further, large positive improvements were observed for a social function. This being a major deficit in ASD (Healy et al., 2018).

A study conducted on 5–8-year-old children in a special school in China showed significant improvements in the social function among children on the spectrum who participated in a 12-week structured PA program (Zhao and Chen, 2018). The program consisted of twice-weekly exercise sessions both lasting 60-min. The intervention program purposely provided opportunities to enhance social interactions in a natural environment conducive to developing communication skills. Participants learned how to interrelate with others and how to express themselves. The program was evaluated quantitatively using Social Skills Improvement System Rating Scales (SISS) and Assessment of Basic Language and Learning Skills—Revised (ABLLS-R) scores as well as qualitative interviews with parents and staff. SISS assessed seven social skills subdomains and significant improvements were seen in communication, cooperation, and self-control. Improvements were also seen in ABLLS-R scores. Parents and volunteer workers gave positive feedback on the program (Zhao and Chen, 2018). Since communication and social interaction are the major deficit areas seen with ASD this study provides evidence of the appropriateness of structured PA programs in the management of ASD with children. The improvements observed could neither be solely attributed to PA itself nor the mode of delivery of the program i.e., were the improvements the result of the physical excursion or team-based activities centered around social interaction? This may be an important area for further exploration. Although this study reported improvements in social interactions which the authors attributed to PA, it should be noted that the design and structure of the program were based upon the TEACCH model. The reported improvements may have resulted from the program used, regardless of the PA components that were incorporated as mere details into a well-established intervention. TEACCH is one of the most validated interventions used to treat children with ASD (Panerai et al., 2002) and is particularly effective in the areas of social and maladaptive behaviors (Virues-Ortega et al., 2013). The TEACCH program is based largely on activities which are normally educational or life-skills. However, these could easily be replaced by activities involving physical exercise. These activities are organized within structured elements of a specialist physical environment, predictable sequence of activities, routines with flexibility, structured work/activity systems, and visually structured activities (Myers and Johnson, 2007). While the results of this 12-week study are promising, there were no long-term assessments. Evaluations were carried after completion of the 12-week intervention only. There is a need for follow-up data and more investigations with longitudinal study-designs in this area. Other studies have shown that vigorous rather than moderate exercise is more beneficial in reducing maladaptive and stereotypic behaviors (Kern et al., 1984; Elliott et al., 1994; Celiberti et al., 1997). This suggests that children with ASD may have a higher threshold to overcome before positive effects begin to appear relative to TD children.

A systematic review (Lang et al., 2010), explored 18 PA studies involving adults and children, all of which reported improvements in either: behavior, academic performance, or physical fitness. However, the authors recommended the development of stronger experimental designs for future studies (Lang et al., 2010). This was reiterated more recently in a similar review (Ruggeri et al., 2020).

## Parental Involvement in Physical Activity

It is now recognized that parents play a paramount role in almost all treatment modalities for ASD. Parents were once considered the cause of ASD. However, they are now considered the most important resource and the most effective factor in promoting behavioral changes in the child with ASD (Schopler, 1987). The irony of this role reversal is that currently numerous therapeutic interventions for ASD are hinged upon the pivotal position of parents as therapists or co-therapists (Panerai et al., 2002). Early interventions have been shown to have a moderate-to-large effect on outcomes among children with ASD (Virués-Ortega, 2010; Virues-Ortega et al., 2013; Beaudoin et al., 2014). Interventions specifically designed to include parents have significantly improved outcomes and have shown to be more intensive (Burrell and Borrego, 2012). Meta-analysis show there to be greater improvements in language-understanding and ASD characteristics when parental involvement was incorporated into interventions (Oono et al., 2013). The equipping of parents with development-enhancing strategies while engaged with their children is considered an essential constituent of these interventions (Landa, 2018). This is concerning interventions in general. Parental support and involvement also appears to be a major component of PA-based interventions and keeping ASD individuals physically active (Nichols et al., 2019). Parental involvement has been shown to augment treatment approaches leading to more strongly positive outcomes (Mendlowitz et al., 1999; Lakin et al., 2004). Lakin et al. showed that PA delivered with family involvement resulted in better outcomes relative to therapy delivered without family involvement (Lakin et al., 2004).

Parental influence on PA with children with ASD also remains a vital area of study concerning young adults on the autism spectrum, especially those of post-secondary education age. These individuals rely heavily on either their parents or caregivers (Hewitt et al., 2017). One study suggested that the strong positive effects of parental involvement in PA were merely coincidental (Burrell and Borrego, 2012). This suggestion highlights the need for further research in the area of parent involvement in PA. Especially since PA appears to be crucial for young adults with ASD (Nichols et al., 2019). ASD children are considered relatively dependent on others for assistance in everyday life (Schall et al., 2014). ASD adolescents are more likely to have “poorer health profiles” and develop chronic diseases as compared to TD individuals. Therefore, it is imperative to identify factors, such as PA, that influence health-states to construct interventions to help young adults with ASD (Warren et al., 2012).

According to Brustad, parents who enjoyed participating in PA positively impacted their children with ASD by encouraging them to take part. Moreover, this also in turn influences ASD children’s capability and therefore enhances their level of engagement (Obrusnikova and Miccinello, 2012). It has also been shown that lower activity scores correlated with having single-parent families. This suggests that two-parent families may result in better outcomes (Memari et al., 2015). We conclude that parental involvement in therapies or interventions lead to greater and more effective outcomes and should be considered whenever applicable. We suggest that this topic of PA and parental involvement should be researched further in both children and adolescents.

## Implementing Physical Activity as An ASD Treatment

Available treatments for ASD focus on making the child with ASD more independent and maximizing their quality of life. This is achieved by minimizing the core characteristics associated with ASD. Thus, the amelioration of maladaptive behaviors is a common management strategy in programs (Landa, 2007). It is apposite to perceive the role of PA as a component of wider more extensive approaches to treating ASD. This broader base involves educational and behavioral multimodal interventions represented by the *comprehensive treatment models*, such as TEACCH, and the *focused intervention practices* represented by certain pharmacological and behavioral interventions (Wong et al., 2015). These focused interventions address the symptoms associated with impaired social skills and communication skills and repetitive and stereotypical behavior patterns and interests. PA may be categorized as one of the focused interventions and implemented as an adjunct to other focused interventions or as a constituent of a comprehensive treatment model (Smith, 2013). Several comorbid symptoms are commonly associated with ASD which include: self-injury, impulsivity, decreased attention, anxiety, depression, and sleep disruption (Levy et al., 2009). Well-established treatments exist, both pharmacological and behavioral, for some of these comorbid symptoms. Psychotropic drugs are utilized to manage irritability, hyperactivity, and repetitive behaviors (Kaplan and McCracken, 2012; DeFilippis and Wagner, 2016). PA may be co-administered with interventions used to treat the listed common comorbidities.

It is of utmost importance that each child receives an individualized treatment plan that targets their needs and allows for the participation of parents, teachers, and other caretakers (Hyman et al., 2020a,b). Tan et al. (2016) found the benefit of PA in children with ASD to be similar to those seen in TD children. There now exist guidelines that allow different sports and educational establishments to implement programs suited for individuals with ASD (Srinivasan et al., 2014). Whether or not these exercises are effective with individuals with ASD of all ages is yet to be determined. Cameron et al. suggested that movement-based interventions that are effective with older children with ASD may not be effective for preschool children (Cameron et al., 2020).

In addition to the guidelines, there are various ways of implementing PA programs. Bremer et al. (2016) considered the two best forms of physical intervention to be martial arts and horseback riding, whilst there was a limited benefit in yoga, dance, and swimming. In contrast, DeJesus et al. (2020) considered dance to positively affect children and improved many of their symptoms, such as social behaviors, communication skills, and psychological wellbeing. This disparity in interpretation may be due to a larger amount of available data for interventions such as martial arts and limited data for dance. Hence, more research is required in the area of dance as an intervention.

An individual with ASD derives benefits from PA in two main ways. Firstly, the impact of PA on weight gain and obesity and secondly reducing maladaptive behaviors. Obesity is a major concern in children with any form of developmental disabilities (Srinivasan et al., 2014). However, children with ASD are at particularly high risk for obesity (Curtin et al., 2014). It is essential to recognize obesity as it is an established comorbid in children with ASD (Pan, 2008; Sowa and Meulenbroek, 2012). This may be due to lack of structure in nutritional intake, over usage of television to calm them, and side-effects of medication (Must et al., 2017). Another contributing factor is that the ASD child might not have a supportive environment (Pan, 2008). The second perspective relates to behavioral elements such as physical condition, self-esteem, and social skills. A major focus has been placed upon improvements in stereotypical behavioral patterns and general social behavior and function (Zhao and Chen, 2018). Studies have shown that improved motor skills positively impact social skills, in addition to reducing stereotypical behaviors (Iliadis and Apteslis, 2020). Therefore, to maintain the health and wellbeing of ASD children, it is essential to include physical activities in their daily life. Several studies show that PA levels in ASD and neurotypical children are significantly different (Pan, 2008; Sowa and Meulenbroek, 2012). Bandini et al. (2013) found that 43% of TD children participated in a 60-min moderate and vigorous PA as compared to 23% of children with ASD.

To facilitate building a PA program for children with ASD, it is important to address multiple factors to decide the most appropriate elements of the program. We here address two issues: (a) *Individual vs. group intervention*; and (b) *Organization of the Program*.

## Individual Vs. Group Intervention

When tailoring a PA program for children with ASD, an essential question should be asked; would an individual-based or a group-based intervention promote larger improvements? PA programs organized within a group situation (team-mates, peers, coaches, and teachers) may enhance development among children with developmental disabilities (Rinehart et al., 2018). The implementation of group-based PA in a social context would offer opportunities for social interactions. Group-setting should facilitate social behavior and communication. However, meta-analysis compared responses to PA delivered as individual-based and group-based interventions and found greatly improved social skills and attenuated maladaptive social behaviors with the individual-based approach (Sowa and Meulenbroek, 2012). Paradoxically, social skills were less improved in a social scenario. Sowa and Mulenbroek concluded that individual-based interventions offer more specific programs and decreased stress levels due to the unpredictable events associated with group activities (Sowa and Meulenbroek, 2012). The individual approach protects the child with ASD from negative emotions arising from being misunderstood by group members (Pan, 2009), and also from tensions between teammates or opponents (Sowa and Meulenbroek, 2012). Another meta-analysis study provided evidence supporting the use of group-based PA programs. Qualitative and quantitative evaluations of group-based PA concluded that programs provided opportunities for social skill development. This study included several modes of group-based activities but only one “team sport” program. The authors suggested a future focus on team sports as a key research area (Howells et al., 2019). There are clearly advantages and disadvantages to group-based activities which must be taken into consideration for the child with ASD with very specific characteristics and behaviors. The notion of highly individualized education programs for children with ASD is now wildly accepted as each child has a unique presentation of core ASD and comorbidity symptoms. Although counterintuitive, the highly individualized program delivered in isolation of the group would seem to be advantageous with respect to social skills development for children with ASD.

## Organization of The Program

When structuring a program for ASD children, it is important to simplify the structure of the program to not overwhelm any participant or their teachers and parents.

Schultheis et al. (2000) modified the TEACCH program to create a recreational program. The new, modified version included three portions: (a) physical structure; (b) schedules, and (c) task organization. By keeping all three aspects in mind they were able to create simplified ways to facilitate introducing physical activities by introducing a schedule with pictures or color codes and a structure of the gymnasium with room divider boundaries. Additional equipment (timers etc.) may also be used as desired (Schultheis et al., 2000). Meta-analysis indicates that TEACCH has moderate-to-large improvements in social behavior and maladaptive behavior, which makes it an ideal program to achieve improvement within these two combined fields of ASD and PA (Virues-Ortega et al., 2013).

It is now well established that PA leads to improvements in multiple domains relevant to ASD. However, research is still needed in this field to refine and improve programs and modes of delivery of programs to maximize the benefit for individuals with ASD (Sorensen and Zarrett, 2014).

## Duration of Positive Effects

PA has been shown to improve classroom behaviors and improve aspects of academic performance in neurotypical children (Álvarez-Bueno et al., 2017). It is self-evident that there are long-term benefits for any individual that achieves academically during childhood. Regarding autistic children, it has been reported that children with ASD who underwent a 14-weeks training program in karate exercise-routines *kata* (choreographed movements performed with technical precision in set sequences) showed a significant reduction in communication deficit and improvements in stereotypic behaviors compared to neurotypical controls. Interestingly, these improvements were observed for up to 1 month after completion of the intervention (Bahrami et al., 2012, 2016). This suggests that learning these karate specific exercise-routines and undergoing the physical exertion that accompanies them has long-lasting effects. These long-term effects may be related to the use of training with traditional martial art rather than PA *per se*. A study with neurotypical school students showed that participating in school-based martial arts (Taekwondo; including deep-breathing and relaxation techniques) training resulted in improved self-regulatory skills (Lakes and Hoyt, 2004). Traditional martial arts have elements of self-awareness and concentration which are not found in standard physical education classes. Adopting PA in conjunction with self-awareness in the formative years may have long-lasting effects. It has been suggested (Diamond, 2015; de Greeff et al., 2018) that cognitively and coordinately demanding physical exercises may be better at improving executive function in children rather than pure exercises and movement (Wang et al., 2020). We consider this an important area of future research for ASD; in the last decade, there has been great interest in studying the relationship of PA with cognitive functioning for neurotypical children (Lees and Hopkins, 2013; Donnelly et al., 2016).

Impaired social and communication skills associated with ASD may lead to social isolation or withdrawal (Bellini et al., 2007), and most interventions work to counter this. Any intervention that addresses social and communication skills should have long-term implications. Most organized sports activities provide environments conducive for the natural building of connections between participants. Sports and games increase opportunities for social interactions. Inherent elements of most sports and games are turn-taking, cooperative play, partnering, exchanges, and general teamwork. All these elements would practically improve both verbal and non-verbal communication skills as well as ensuring social engagement. Activities with a structured program would also have interactions between children and their teachers. A program of structure sporting activities would be a natural area in which to implement the very tenants of established ASD therapies such as TEACCH (Zhao and Chen, 2018). These therapeutic approaches all have the same goal of having a life-long influence on the child with ASD.

## Innovative Ways to Introduce PA to Children with ASD

There are challenges faced by individuals with ASD regarding performing certain exercises. Therefore, research has concentrated on innovative ways of introducing PA to children and adolescents with ASD. One such innovation in the delivery of PA is *Exergaming*.

While still a new field of study, there has been a growing interest in finding different ways to include PA in the lives of autistic individuals and overcoming the challenges that exist in a normal PA program. For example, some findings have indicated that *Exergames* can be a potential tool to treat both children and adolescents with ASD. *Exergames* are defined as any game that has a combination of video games and physical interaction with participants online. Its advantages include being more enjoyable and playful, which may lead to an increase in adherence. Lima et al. (2020) have shown the benefits have been perceived in fitness perspectives only. More studies are needed in this field to cover the effect of *Exergaming* on stereotypical behaviors, social and cognitive skills.

## Conclusions

PA is effective as a therapeutic strategy for the management of ASD. However, there is a paucity of research evidence about the long-term effects of interventions based upon PA. There is a difference of opinion as to the detailed nature of the activities and exercises that compose an effective PA program. There is also a need for refinements in program delivery methods, which is indicative of the actual autistic condition itself. Each person affected by ASD has a highly individualized set of symptoms and characteristics for which a highly individualized therapeutic program is warranted. The development of workable programs requires research and evidence gathering to produce generalized guidelines. These guidelines may come to govern very specialized structured and individualized interventions for children and adolescents with ASD.

## Author Contributions

The abstract was written by JS and edited by SF. The introduction was written by SF, followed by the general benefits of exercise by JA. “The Benefits of Physical Activity in ASD” section was written by JS. ZS wrote the “Parental Involvement in Physical Activity” section. SA-S explained how to implement physical activity as an ASD treatment by looking at Group vs. Individual Interventions and Organization of the Program. SF has also elaborated on the duration of positive effects, and “Innovative Ways to Introduce PA to Children With ASD” section. JS also wrote the conclusion. SF has also managed references using endnote and helped JS in editing the article as well as doing the final review. The article was also reviewed by ER, who additionally contributed ideas and added suggestions to the article.

## Conflict of Interest

The authors declare that the research was conducted in the absence of any commercial or financial relationships that could be construed as a potential conflict of interest.
